# Supplementary material for: Harnessing clinical annotations to improve deep learning performance in prostate segmentation
Source: PLoS One. 2021 Jun 25;16(6):e0253829. doi: 10.1371/journal.pone.0253829 (PMC8232529; doi:10.1371/journal.pone.0253829)
Supplement: S1 Table — Full acquisition data is not available for the PROMISE12 dataset, and the counts for images acquired at different field strengths and resolutions are not available. (DOCX) [file pone.0253829.s001.docx]

**S1 Table. Imaging acquisition parameters for study datasets.**

|  | UCLA (*n*=1620) | ProstateX-2 (*n*=99) | PROMISE12 (*n*=50) |
| --- | --- | --- | --- |
| Vendor(s) | Siemens | Siemens | Siemens, GE |
| Field Strength | 3T | 3T | 1.5T, 3T |
| In-plane resolution (mm) | 0.664 | 0.5 | 0.25-0.75 |
| Slice thickness (mm) | 1.5 | 3.6 | 2.2-4.0 |
| TR (ms) | 2200 | 5660 | Not available |
| TE (ms) | 201 | 104 | Not available |
| Endorectal coil used (*n, %*) | 29 (1.8%) | 0 (0%) | 24 (48%) |

Full acquisition data is not available for the PROMISE12 dataset, and the counts for images acquired at different field strengths and resolutions are not available.
