# Supplementary figures and images for: Harnessing clinical annotations to improve deep learning performance in prostate segmentation
Source: PLoS One. 2021 Jun 25;16(6):e0253829. doi: 10.1371/journal.pone.0253829 (PMC8232529; doi:10.1371/journal.pone.0253829)

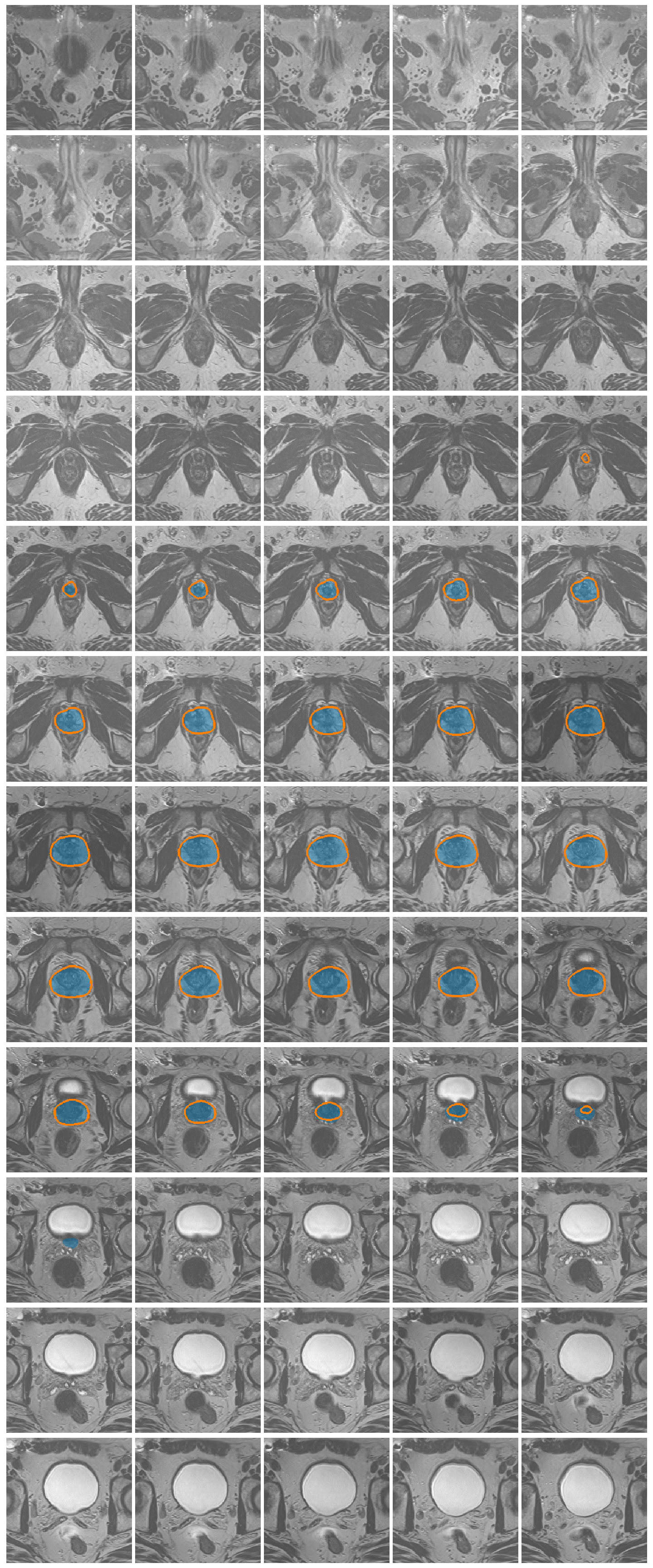

Supplement: S1 Fig — Orange contour depicts ground truth segmentation. Shaded blue area depicts model segmentation. Slices depicted from apex to base. The soft Dice coefficient for this sample was 0.928, and the average Hausdorff distance was 0.085. (PNG) [file pone.0253829.s002.png]

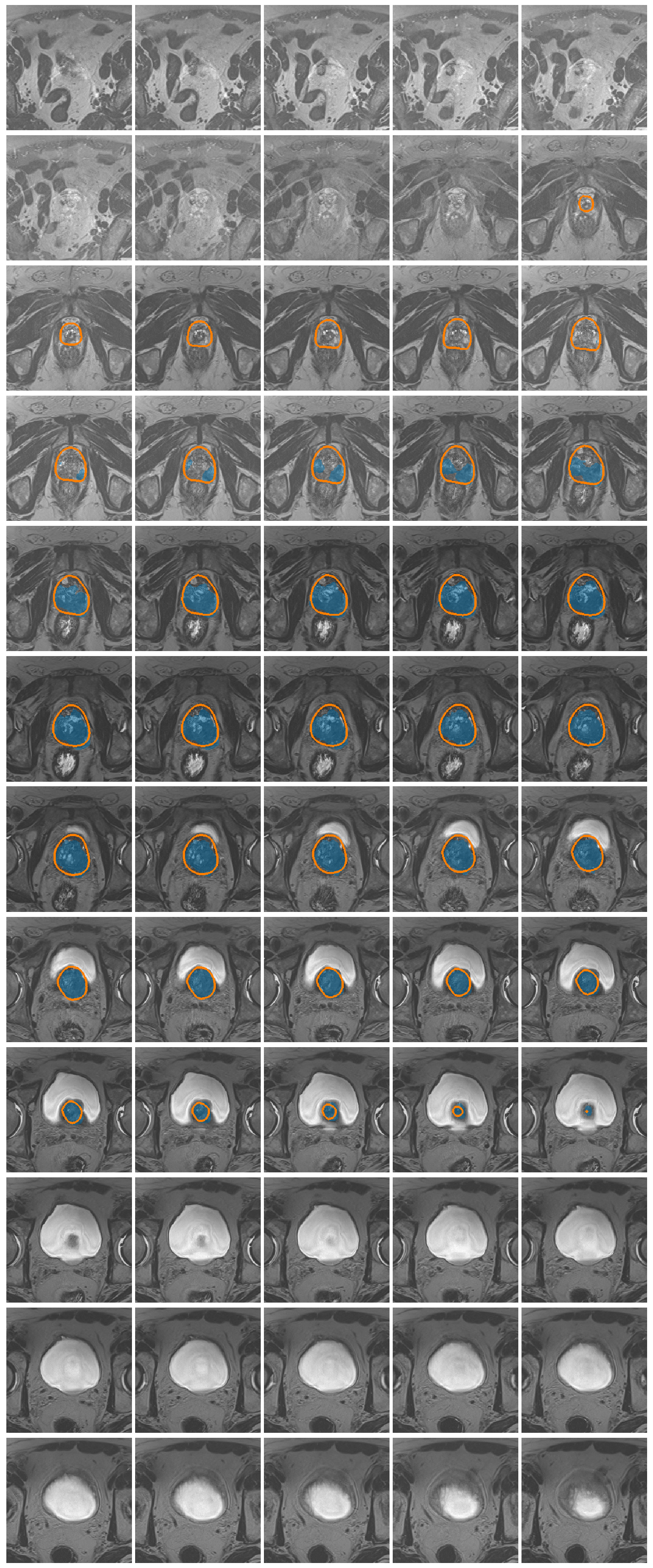

Supplement: S2 Fig — Orange contour depicts ground truth segmentation. Shaded blue area depicts model segmentation. Slices depicted from apex to base. The soft Dice coefficient for this sample was 0.738, and the average Hausdorff distance was 0.935. (PNG) [file pone.0253829.s003.png]

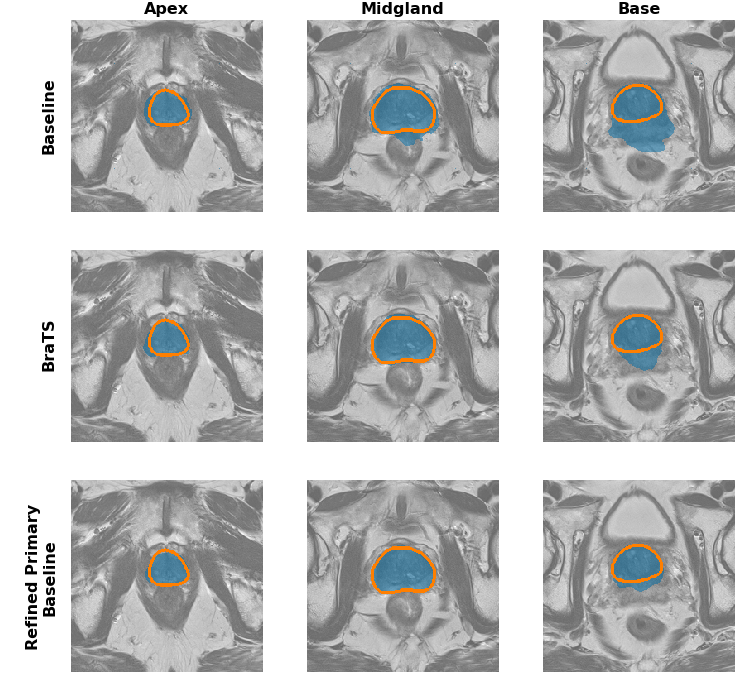

Supplement: S3 Fig — Orange contour depicts ground truth segmentation. Shaded blue area depicts model segmentation. The soft Dice coefficient and average Hausdorff distance metrics were 0.645 and 1.024 for the baseline model, 0.864 and 0.167 for the BraTS model, and 0.932 and 0.079 for the refined primary baseline model. (PNG) [file pone.0253829.s004.png]

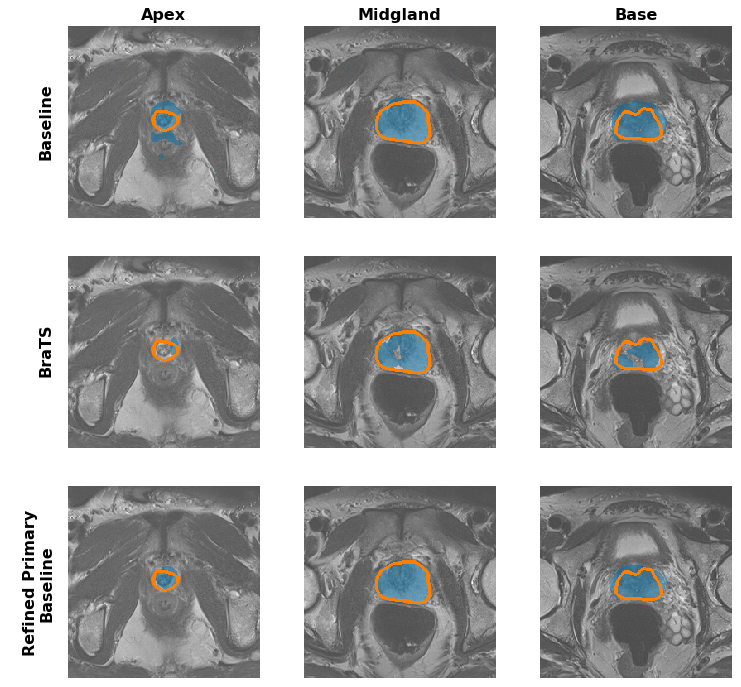

Supplement: S4 Fig — Orange contour depicts ground truth segmentation. Shaded blue area depicts model segmentation. The soft Dice coefficient and average Hausdorff distance metrics were 0.536 and 2.974 for the baseline model, 0.678 and 0.291 for the BraTS model, and 0.910 and 0.102 for the refined primary baseline model. (PNG) [file pone.0253829.s005.png]
