# Supplementary material for: Harnessing clinical annotations to improve deep learning performance in prostate segmentation
Source: PLoS One. 2021 Jun 25;16(6):e0253829. doi: 10.1371/journal.pone.0253829 (PMC8232529; doi:10.1371/journal.pone.0253829)
Supplement: Attachment — Submitted filename: Response to Reviewers.pdf [file pone.0253829.s006.pdf]

Response to Reviewers
(point-by-point)

Thank you for the very helpful review of our work. Based on your suggestions, we have made numerous improvements to our paper, and have detailed improvements made in response to your comments below:

Abstract
1. Materials and method: It could be relevant to mention that 5-fold cross-validation was performed, possibly also mentioning the initial train-validation split in the abstract.

We have added information about the cross-validation split and clarified that the evaluation scores are calculated across the full dataset to the section.

2. Results: Please clarify whether the dice score of 0.91 was obtained on the training or independent testing data. It appears that all of the data was used for model training and there was no independent test set on the UCLA data.

We have added a parenthetical clarification to the section that the results are across CV folds.

Introduction
3. Paragraph 1 sentence 1: I recommend either keeping and using the abbreviation for prostate cancer (PCa) consistently throughout the paper or removing the abbreviation.

We have removed this abbreviation from the paper for clarity.

4. Paragraph 1 sentence 3: At some institutions, urologists review the segmentations on T2-weighted MRI, whereas other institutions use radiologists or a mix.

We have updated this sentence to more accurately represent the process.

5. Paragraph 1 sentence 4: Please change Phillips to Philips. This typo is repeated elsewhere as well.

We have fixed the spelling of “Philips” throughout the paper.

6. Paragraph 1 sentence 4: If one exists, please provide a citation for DynaCAD Prostate (Philips) segmentation.

Philips has not published any work on the performance or operation of DynaCAD, however we have cited the product website.

7. Paragraph 2 sentence 1: This makes a definitive assumption that inaccurate segmentations are sufficient for clinical tasks. Please provide a citation that supports this argument. Otherwise, I would not state this so definitively. For accurately targeting cancer in the prostate it is not the volume that is most important, but the relationship of the cancer to the edge of the prostate.

We have removed this assertion from the paragraph to put emphasis on the fact that accurate segmentations are critical for downstream image analysis, such as cancer detection.

8. Paragraph 3: It would enhance the paper if the authors could provide a range in the number of cases that have previously been utilized for training different prostate segmentation models and cite the models; not all prostate segmentation models have been trained on small datasets, e.g.,:
"Data Augmentation and Transfer Learning to Improve Generalizability of an Automated Prostate Segmentation Model. Thomas H. Sanford, Ling Zhang, Stephanie A. Harmon, Jonathan Sackett, Dong Yang, Holger Roth, Ziyue Xu, Deepak Kesani, Sherif Mehralivand, Ronaldo H. Baroni, Tristan Barrett, Rossano Girometti, Aytekin Oto, Andrei S. Purysko, Sheng Xu, Peter A. Pinto, Daguang Xu, Bradford J. Wood, Peter L. Choyke, and Baris Turkbey. American Journal of Roentgenology 2020 215:6, 1403-1410"

We have added citations for a sample of previously published prostate segmentation models that use challenge datasets and that use institutional datasets, as well as a range for the sizes of the datasets used.

Materials and methods
9. Primary dataset: If possible, please mention or describe whether scans were compliant with PIRADS specifications.

Our dataset begins in 2010 which predates PI-RADS. As such, we chose to use the T2 SPACE protocol images for our UCLA dataset, as the parameters remained the same through the collection period. We have added additional information about our acquisition protocol and parameters for the T2 images to the Materials and Methods section and table S1; we have also added information about the challenge datasets to table S1.

10. Primary dataset: To help readers assess generalizability, please describe the distribution between scans obtained on different scanners (e.g., Philips, GE, and/or Siemens).

We have clarified in the Materials and Methods section and table S1 that all primary dataset scans were performed on Siemens scanners.

11. Primary dataset: Please include whether scans were acquired using endorectal coil or not? Did this effect model performance?

An endorectal coil was used for a small fraction (<2%) of patients. We have noted this information in table S1. Because such a small number of patients had an endorectal coil, there is not enough data to conclusively determine the impact of the coil on model performance. For the patients in the primary dataset who were imaged with an endorectal coil, mean overall Dice coefficient was 0.884 (vs. 0.909 across the whole dataset) and mean AHD was 0.200 (vs. 0.156 across the whole dataset); both values are within one SD of the whole dataset means.

12. Model, training and evaluation: Consider calculating the Hausdorff distance in all cases in the internal test set to demonstrate how gland segmentation accuracy may impact the location of the target.

We have added the average Hausdorff distance as an evaluation metric and reported our findings as they relate to model performance improvement throughout the paper, tables, and figures.

13. Model, training and evaluation: The authors might want to mention that the results were reported as mean +/- standard deviation.

We have added additional clarity in this section regarding how the evaluation metrics were computed across all images in the dataset.

14. Baseline models: What was the initial training-validation split in the 5-fold cross-validation?

We have added additional clarity regarding the training/validation set split to the Baseline models section.

15. Please make it clear who performed the expert segmentations used as the gold standard for model training and for calculating dice scores.

We have clarified that the segmentations were performed clinically by the attending radiologist for the case at the time of the initial imaging study; most of the radiologists are not authors of this paper.

Results

16. It appears that the Dice of 0.91 was obtained from the 5 fold cross validation rather than from a held out test set in the UCLA data or from testing in the external dataset. Is this correct? If so, why was testing not performed in a held out test set from the UCLA data?

The mean overall Dice and AHD statistics reported on the primary dataset are the mean over the study-level scores for every image in the dataset included in the evaluation. These scores were calculated using the cross-validation model that was trained without that particular image in the training set (i.e. the image was in the held-out fold for that model). Typically studies require a held-out test set to address the potential bias

introduced by model-level optimizations (i.e. adjusting the model design to optimize performance). However, because our study design involved "freezing" the model configuration in order to have the only dependent variables in our experiments be data-related, there was no need for a separate held-out test set because there is no risk of bias introduced from model optimization over the course of the experiments. As such, we chose to use all of the available data in the evaluation in order to provide a fairer assessment of model performance across a larger set of images. We believe this provides the best estimate of model performance and variance.

17. Please report the results as mean +/- standard deviation.

We have updated the paper throughout to report results in this way.

18. The authors mention that the model performs better after refinement. Why is the performance so poor prior to refinement (Dice 0.465)?

The PX2 dataset was acquired with homogeneous imaging parameters, and the P12 dataset was acquired with very heterogeneous imaging parameters. However, the parameter range for the P12 dataset does overlap more closely with the UCLA dataset. We believe this explains why the UCLA model before refining does better on P12 than PX2, and after refining, the PX2 model is superior because of the internal consistency. Unfortunately, because individual sample acquisition parameters were not provided with the P12 dataset, it is not possible to run an analysis to determine whether this hypothesis is correct.

19. Is the improvement in dice clinically significant (i.e. does it make the cancer target location more accurate)?

Unfortunately, it is not possible for us to evaluate the impact of the performance improvements on biopsy targeting due to the retrospective design of our study, which does not include access to the real-time ultrasound used for image fusion. We have added a note to the limitations section of the paper about this and to highlight a prospective evaluation of targeting as future work.

Discussion
20. There appears to be a discrepancy between the results in the abstract and what can be found in the discussion section. Did using the model trained on the large internal dataset as a pre-trained template increase performance (dice score) by 39% and 49% in the two respective external datasets – or was it 30% and 49%?

We have corrected this error (the correct proportions are 30% and 49% improvements in Dice).

21. To help put this into context, I'd recommend citing other prostate segmentation papers that perform worse, equal to, and better than your model.

We have added comparisons and citations to the leading prostate segmentation models on challenge and private datasets to facilitate easier comparison of our results in context to the discussion section of the paper. Since our results are comparable to the best published results available, we provided one comparison for each group in the discussion, and added a series of additional citations for models trained on challenge or private datasets to the introduction section of the paper.

22. In calculating the dice in the clinical dataset, what was used as the gold standard segmentation? If it just the clinical segmentation, is it possible that the model segmentation is better than the gold standard?

For the primary dataset, we used the clinically generated segmentation. As such, it is possible that some differences between model predictions and the ground truth are the result of inaccurate labels, rather than model error. We have added a note to the limitations section about this possibility.

Tables and figures:
23. It should be clear to the reader that the results are shown as mean +/- standard deviation.

We have added this note on each table and to the beginning of the results section text for clarity.

24. Space permitting, a table that shows relevant MRI characteristics and dataset composition would enhance the paper's value (e.g., it could include the number of cases, scanners, MRI sequence, magnetic field strength, slice thickness, in-plane resolution).

We have added this information as table S1.

25. There should be figures that demonstrate the performance of the model visually.

We have added a number of figures (as described below) to help demonstrate our model's performance and results.

26. It would be helpful if the paper provided an overview diagram of the 3D U-Net architecture and possibly also the preprocessing steps in the same diagram.

We have added such a diagram as Figure 1.

27. I recommend including 1-2 figures demonstrating representative test cases from the internal and external datasets, showing every slice from base-to-apex, including segmentation outputs before and after refinement.

We have added two “every slice” figures demonstrating segmentation outputs as figures S2 and S3. We have also added a figure with representative slices as figure 2. Finally, we have added figures S4 and S5 which depict representative segmentation slices of the same prostate for the baseline, BraTS, and refined primary baseline models.

28. It would be great to see the addition of box plots (or similar plots) that show dice score distributions (range, IQR, median) before and after refinement.

Based on your suggestion, we looked at a number of potential plots (including box plots and violin plots) and found that strip plots best presented the information. We have added strip plots for Dice and average Hausdorff score for the challenge datasets across the baseline and refined models as Figure 3

29. It would be interesting to see whether the choice of deep learning model affects the results significantly, more so than the size of the dataset. One idea could be to look at ImageNet or the holistically nested edge detector network (https://www.ncbi.nlm.nih.gov/pmc/articles/PMC5565676)

We agree that exploring the choice of deep learning model would be of great interest. We chose to fix our model parameters for the purposes of this study in order to focus our analysis on the impact of data size and relevance; however in future work we would like to further study the impact of model design on this outcome. This limitation is noted in the limitations section of the paper.

Limitations section:
30. I recommend incorporating the following into the limitations.
a. The 0.91 dice reported in the abstract is not from a held out test set or the external testing.

We have updated the limitations section to clarify why we held the model design constant and included data from all folds in the valuation, rather than a held-out test set. We have also included performance metrics for the challenge datasets in the abstract as well.
